# Oral Health, Hygiene, and Nutrition in Individuals with Disabilities: Comparing Institutional and Family Living Environments

**DOI:** 10.1111/scd.70266

**Published:** 2026-09-26

**Authors:** Zoumpoulakis Michail, Zouloumis Lambros, Venetis Grigorios, Tzanakis Nikolaos

**Affiliations:** ^1^ Department of Oral and Maxillofacial Surgery School of Dentistry Aristotle University of Thessaloniki Thessaloniki Greece; ^2^ Department of Oral and Maxillofacial Surgery School of Dentistry Aristotle University of Thessaloniki Thessaloniki Greece; ^3^ School of Medicine University of Crete Heraklion Crete Greece

**Keywords:** community, disability, institution, nutrition, oral health, oral hygiene

## Abstract

**Aims:**

Individuals with disabilities (IwD) often face challenges affecting oral and nutritional health, potentially influenced by their living environment. This study aimed to assess the relationships between oral health, hygiene, and nutrition among IwD in institutional and community settings.

**Methods & Results:**

A cross‐sectional study was conducted among IwD residing in institutions and the community. Data collection included structured questionnaires, clinical dental indices, oral function indicators, and anthropometric measures. Statistical analysis was performed using Stata18 with significance set at *p* ≤ 0.05. A total of 207 IwD were examined—105 institutionalized (mean ± SD age = 60.37 ± 18.19) and 102 community dwellers (65.53 ± 17.83). Both groups showed high dental treatment needs and compromised nutritional status. Institutionalized participants had poorer oral hygiene, higher caries burden, and greater caregiver dependence, whereas community dwellers exhibited better oral health but less favorable nutritional profiles. Significant associations were identified between anthropometric and oral health indicators.

**Conclusions:**

IwD exhibit poor oral health and hygiene overall. Living environment influences oral and nutritional outcomes, with multiple interrelations between them. Multidisciplinary interventions with caregiver support are essential, while longitudinal studies are needed to clarify causal links between diet and oral health.

## 1 Introduction

Oral health is an integral component of general health and well‐being, yet it is frequently neglected in individuals with disabilities (IwD). This population faces multifactorial challenges in maintaining oral health, including motor and cognitive impairments, limited access to dental care, and heavy reliance on caregivers. As a result, they often present with higher prevalence of oral diseases and inadequate daily oral hygiene compared with the general population [1, 2, 3, 4, 5, 6, 7].

Additionally, impaired masticatory function—whether due to tooth loss, reduced number of occluding pairs, or poorly fitting prostheses—has been shown to restrict food choices and compromise nutrient intake [8, 9]. Studies consistently report that individuals with fewer natural teeth and inadequate prosthodontic rehabilitation exhibit poorer dietary diversity and nutritional status, while maintaining a functional dentition or using well‐fitting prostheses is associated with improved nutrition and overall health [8, 9, 10, 11, 12]. On the other hand, poor nutritional status has been linked to adverse oral outcomes [13]. Malnutrition in general has been associated with several conditions that can cause oral pain or oral discomfort [14, 15]. Underweight status has been associated with greater tooth loss, compromised masticatory efficiency and greater caries susceptibility. These oral impairments can lead to dietary restrictions that perpetuate nutritional deficiencies [13, 16]. Conversely, obesity has been correlated with caries development [7, 17, 18]and xerostomia [12].This bidirectional relationship between oral health and nutrition has been well highlighted in the literature, whereby poor oral health can both contribute to and result from inadequate nutritional status. This fact is particularly relevant in IwD, where the various functional, cognitive, and behavioral limitations may further exacerbate the impact of poor oral status on food intake and vice versa [12, 19, 20, 21].

The high functional dependency, typically present in this group, can further complicate this interaction. Lower scores on functional or independence indices have been linked to poorer oral hygiene practices, reduced dietary diversity, and increased reliance on caregivers for daily self‐care, as consistently reported in institutionalized or dependent populations [7, 12]. Recent evidence also indicates that impaired functional ability itself is an important determinant of poor oral hygiene and oral health, as demonstrated in home care clients [2]. Similarly, lower cooperation level has been linked to worse oral health outcomes [5, 6].

Living environment is another determinant of both oral health and nutritional status. Residents of institutions often have fewer remaining teeth, worse oral health and hygiene, and more extensive unmet dental treatment needs, due to limited access to individualized dental care, lower frequency of preventive interventions and restricted dietary variety compared to community‐dwelling peers [1, 10, 21]. Community‐dwelling IwD may benefit from more personalized dietary choices and family‐supported oral care, whereas institutionalized individuals are often subject to standardized meal plans, texture‐modified diets, and limited supervision of daily hygiene [7, 12, 21]. Studies have reported that institutionalized populations often present higher sugar consumption, increased meal frequency, and poorer toothbrushing practices, which may contribute to calculus accumulation and caries risk [1, 13]. Importantly, these risks can persist even in individuals with low Body Mass Index (BMI), as underweight status has also been associated with poorer oral health outcomes and greater tooth loss [15, 16]. Evidence on ultra‐processed foods also suggests that diets dominated by sugar‐rich, soft and pureed meals—frequent in institutional settings—may increase the risk of both poor nutrition and oral disease [13, 22, 23].

Despite the recognized associations, previous studies have typically evaluated oral or nutritional outcomes in isolation [11, 15] and often relied solely on BMI as a proxy for nutritional status [11, 16, 17, 18] However, BMI does not adequately reflect fat distribution or body composition. Measures such as triceps skinfold thickness (TSF) and waist circumference (WC) provide complementary information regarding adiposity and metabolic risk [24, 25]. To our knowledge, there is an important gap in the literature as few studies have simultaneously integrated detailed dental indices, anthropometry, and functional/behavioral characteristics in IwD while also comparing institutional and family living environments and, to date, no comparable study has been conducted in the Greek population. Understanding these interrelationships may inform targeted clinical, caregiving, and institutional policies aimed at improving both oral and nutritional health.

Therefore, the aim of the present study was to evaluate the differences and relationships between oral health, oral hygiene, and nutritional status among Greek IwD, comparing those living in institutional care with those residing in family settings.

## 2 Materials and Methods

A convenience sampling method was used in this cross‐sectional study. 105 institutionalized participants were recruited from one public and two private residential care facilities in the Lasithi Prefecture of Crete in Greece (aged 17–97 years), while 102 community‐dwelling participants (aged 19–99 years) were identified through family caregivers in the same region. All eligible IwD were invited to participate. Randomization and stratified sampling were not employed.

Data was collected through structured questionnaires administered by the investigator via face‐to‐face interviews with participants and/or their caregivers when necessary.

Oral health and hygiene were assessed by a hospital‐based dental professional with clinical expertise in special care dentistry/ hospital dentistry. The clinical examination included evaluation of dental caries experience using the Decayed, Missing and Filled Teeth (DMFT) and Decayed, Missing and Filled Surfaces (DMFS) indices, with detailed recording of their individual components decayed teeth (DT), missing teeth (MT), filled teeth (FT), decayed surfaces (DS), missing surfaces (MS) and filled surfaces (FS) [26]. Oral hygiene status was measured using the Simplified Oral Hygiene Index (OHI‐S) where scores were categorized as good (0.0–1.2), fair (1.3–3.0), and poor (3.1–6.0) [27]. In addition, oral conditions associated with pain or discomfort—such as xerostomia, angular cheilitis, atrophic glossitis, ulcers, gingival hyperplasia, and soft and hard tissue trauma—were examined. Masticatory function was evaluated through the number of natural teeth, posterior occlusal pairs, and use of prosthodontic appliances [28].

Anthropometric nutritional measurements, including BMI, TSF, and WC were performed by a trained professional. Participants were categorized according to WHO criteria as underweight (17.0–18.5 kg/m^2^), normal weight (18.6–24.9 kg/m^2^), overweight/pre‐obesity (25.0–29.9 kg/m^2^), obesity class I (30.0–34.9 kg/m^2^), obesity class II (35.0–39.9 kg/m^2^), and obesity class III (≥ 40 kg/m^2^). Disease risk based on WC measurements was also classified following WHO guidelines: for males — low risk (< 94 cm), high risk (94–101 cm), and increased high risk (≥ 102 cm); for females — low risk (< 80 cm), high risk (80–87 cm), and increased high risk (≥ 88 cm) [24].

The participants’ independence status was assessed using the Katz Index of Independence in Activities of Daily Living [29], their cooperation level was assessed using the Frankl behavioral scale [30]. The Charlson Comorbidity Index (CCI) was used to assess the burden of systemic disease. It is a weighted scoring system in which higher values indicate progressively greater one‐year mortality risk: 12% for a score of 0, 26% for scores of 1–2, 52% for scores of 3–4, and 85% for scores ≥ 5. Thus, higher CCI values reflect substantially impaired general health and reduced long‐term survival probability [31].

The study protocol was approved by the administrative boards of the three participating institutions. Written informed consent was obtained from all participants or their legal guardians, when necessary. Participants identified with urgent dental or oral health needs during examination, were referred to appropriate dental services for further evaluation and treatment.

All statistical analyses were performed using Stata (StataCorp. (2023). Stata Statistical Software: Release 18. College Station, TX: StataCorp LLC TX, USA). Continuous variables were assessed for normality using the Shapiro–Wilk test. Depending on data distribution, comparisons between two groups were conducted using either parametric tests (Student's *t*‐test) or non‐parametric alternatives (Mann–Whitney U test). Categorical variables were compared using the chi‐square test or Fisher's exact test when appropriate. Associations between anthropometric measures and oral outcomes were examined using multivariable regression models. Regression analyses were stratified by living environment (institutionalized vs community‐dwelling) to examine whether associations differed between residential settings.

Linear regression was applied for continuous outcomes (e.g., OHI‐S, DMFT components), whereas logistic regression was used for binary outcomes (e.g., presence of xerostomia or mucosal lesions). Covariates included age, sex, residential status, Charlson Comorbidity Index, independence level (Katz). Variables were included in multivariable models based on clinical relevance and univariate screening (*p* < 0.20). Multicollinearity was assessed using variance inflation factors (VIF<5 considered acceptable). Results are presented as β coefficients or odds ratios (ORs) with 95% confidence intervals. Statistical significance was set at *p* ≤ 0.05 for all tests. Results are presented as means ± standard deviation or medians (Min‐Max) and proportions where applicable.

## 3 Results

A total of 207 IwD were examined— 105 residing in institutions (group 1) aged (mean±SD, yrs) 60.37 ± 18.19 and 102 living with families (group 2) aged 65.53 ± 17.83, with comparable distribution of males and females across the two groups (M/F, 58/47 in group 1 vs 50/52 in group 2). Overall, 25.12% of participants had physical disabilities, 14.98% mental disabilities, and 59.90% multiple disabilities. Multiple disabilities predominated in both groups (62.86% group 1; 56.86% group 2). Among those with a single disability, physical disabilities were more common in the community (25.29%), whereas mental disabilities were more prevalent in institutions (21.90%).

- Differences between the two groups
  - Oral Health and Hygiene
    The indicators reflected high dental treatment needs across both groups, with the overall population presenting a median DMFT of 16.5 {0–28}, a median DMFS of 57 {0–128}, and a median OHI‐S score of 3.25 {0–6}. Furthermore, both institutionalized and community‐dwelling participants demonstrated compromised masticatory efficiency; however, the prevalence was notably higher among institutionalized individuals, 74.29% of whom had fewer than four posterior occlusal pairs, compared with 56.87% in the community‐living group (*p* = 0.007) and fewer natural teeth (Mdn = 16 {0–32} vs Mdn = 21 {0–32}, *p* = 0.0018). When comparing participants by living environment, institutionalized individuals generally exhibited significantly poorer oral health and hygiene outcomes, including higher DT, DS and Calculus and Debris indices, although their FT and FS scores were significantly lower than those of the community dwellers. On the other hand, mean BMI, WC and TSF values tended to be higher among the family‐living participants (Table 1).
    Moreover, institutionalized individuals showed lower independence (30.48% vs 82.35%, *p* < 0.001), greater dependence on caregivers for toothbrushing (85.71% vs 12.75%, *p* < 0.001), poor to non‐existent daily oral hygiene (92.38% vs 18.63%, *p* < 0.001), and fewer annual dental visits (M = 0.19 ± 0.63 vs M = 0.74 ± 0.91, *p* = 0.001). They also reported higher meal frequency (more than five meals per day: 98.10% vs 33.33%, *p* < 0.001) and greater sugar exposure (more than three times daily: 88.57% vs 8.82%, *p* < 0.001).
  - Nutrition and anthropometry
    BMI distribution revealed that the majority of the institutionalized participants were in the normal and pre‐obesity categories, while smaller proportions were classified as obese (Classes I–III) and even fewer as underweight (Table 2). As far as the family living group, the majority were in the pre‐obesity and obesity categories. Waist circumference measurements revealed that a considerable proportion of both men and women from both groups are classified as high or increased high risk for chronic disease development based on their body fat distribution (Table 2). To further illustrate the overall medical complexity of the sample, both groups demonstrated elevated Charlson Comorbidity Index scores (family‐living: M = 4.02 ± 2.45; institutionalized: M = 3.41 ± 2.19, *p* = 0.089), with no statistically significant difference between them.
- Associations between the two groups
  Several statistically significant associations were identified between anthropometric nutritional measures and oral health indicators (Table 3).
  - In the family living participants, BMI was positively associated with DS as well as the number of posterior occlusal pairs, hard and soft tissue trauma, while inverse associations were observed with FT and FS. As for the institutionalized participants, BMI was positively correlated only with soft tissue trauma, while negatively correlated with Calculus and OHI‐S Indices.
  - WC was positively associated with DS and inversely associated with FT and FS, but only in the family living group. It also showed a borderline positive association with DT as well as with the number of posterior occlusal pairs.
  - TSF correlated with the prevalence of xerostomia and soft tissue trauma in the family living IwD, as well as with angular cheilitis in the institutionalized ones.

**TABLE 1:** Comparison of key oral health and nutritional indicators between institutionalized and community‐dwelling IwD.

Variable	Family‐living IwD	Institutionalized IwD	*p* value
Debris Index	1.6 {0.3–3.2}	3.0 {0.5–3.0}	< 0.001
Calculus Index	0.7 {0.0–3.0}	1.7 {0.0–3.0}	< 0.001
OHI‐S	2.3 {0.3–6.0}	4.6 {0.0–6.0}	< 0.001
DT	1.0 {0.0–17.0}	4.0 {0.0–24.0}	0.005
MT	5.0 {0.0–28.0}	4.0 {0.0–28.0}	0.510
FT	4.0 {0.0–18.0}	0.0 {0.0–10.0}	< 0.001
DMFT	18.0 {0.0–28.0}	14.0 {0.0–28.0}	0.319
DS	2.0 {0.0–51.0}	9.0 {0.0–96.0}	< 0.001
MS	24.0 {0.0–128.0}	20.0 {0.0–128.0}	0.455
FS	9.0 {0.0–64.0}	0.0 {0.0–31.0}	< 0.001
DMFS	66.0 {0.0–128.0}	52.0 {0.0–128.0}	0.496
BMI (kg/m^2^)	29.3 ± 6.4	25.5 ± 8.5	0.001
TSF (mm)	22.3 ± 9.3	16.0 ± 7.8	< 0.001
WC (cm)	106.1 ± 13.4	95.0 ± 18.4	< 0.001

*Note*: Comparisons were conducted using either parametric tests (Student's *t*‐test) or non‐parametric alternatives (Mann–Whitney U test). Values presented as mean ± SD for parametric variables and median {min‐max} for non‐parametric variables.

Abbreviations: BMI, Body Mass Index; DMFS, Decayed, Missing, and Filled Surfaces index; DMFT, Decayed, Missing, and Filled Teeth index; DS, Decayed Surfaces; DT, Decayed Teeth; FS, Filled Surfaces; FT, Filled Teeth; MS, Missing Surfaces; MT, Missing Teeth; OHI‐S, simplified oral hygiene index; TSF, Triceps Skinfold Thickness; WC, Waist Circumference;

**TABLE 2:** Distribution of participants according to BMI and waist circumference (WHO classification).

	BMI			Risk of disease based on WC		
Group	Underweight	Normal weight	Overweight / Obese (Classes I‐III)	Low Risk	High Risk	Increased Risk
Family IwD	3(2.9%)	18(17.6%)	81(79.4%)	6(2.9%)	17(8.2%)	79(38.2%)
Institutionalized IwD	9(8.6%)	41(39.1%)	55(52.4%)	39(18.9%)	17(8.2%)	49(23.7%)
Total	12(5.8%)	59(28.5%)	136(65.7%)	45(21.7%)	34(16.4%)	128(61.9%)

Abbreviations: BMI, Body Mass Index; WC, Waist Circumference; values are expressed as *n* (%).

**TABLE 3:** Significant associations between anthropometric nutritional measures and oral health indicators.

Family living Group				
A. Linear regression models				
Outcome	Predictor	β	95% CI	*p*‐value
FT	BMI	−0.136	−0.267 to −0.006	0.040
DS	BMI	0.443	0.070 to 0.816	0.020
FS	BMI	−0.463	−0.875 to −0.051	0.028
DT	WC	0.058	−0.002 to 0.119	0.050
FT	WC	−0.098	−0.159 to −0.038	0.001
DS	WC	0.260	0.085 to 0.435	0.004
FS	WC	−0.278	−0.472 to −0.085	0.005
Posterior occlusal pairs	WC	0.968	0.937 to 1.000	0.050

B. Logistic regression models				
Outcome	Predictor	OR	95% CI	*p*‐value
Posterior occlusal pairs	BMI	0.932	0.871 to 0.997	0.043
Hard tissue trauma (attrition)	BMI	0.931	0.869 to 0.998	0.046
Soft tissue trauma in gums due to biting	BMI	1.152	1.011 to 1.312	0.034
Soft tissue trauma in gums due to biting	TSF	1.130	1.028 to 1.242	0.011
Xerostomia	TSF	1.128	1.051 to 1.210	0.001

Institutionalized group				
A. Linear regression models				
Outcome	Predictor	β	95% CI	*p*‐value
Calculus Index	BMI	−0.041	−0,068 to −0.137	0.003
OHI‐S	BMI	−0.056	−0.104 to −0.007	0.023

B. Logistic regression models				
Outcome	Predictor	OR	95% CI	*p*‐value
Soft tissue trauma in lips due to biting	BMI	0.862	0.766 to 0.971	0.015
Angular Cheilitis	TSF	0.856	0.756 to 0.970	0.015

*Note*: Models adjusted for age, sex, Charlson Comorbidity Index, independence level (Katz index); β: unstandardized linear regression coefficient;

Abbreviations: BMI, Body Mass Index; CI, confidence interval; DMFS, Decayed, Missing, and Filled Surfaces index; DMFT, Decayed, Missing, and Filled Teeth index; DS, Decayed Surfaces; DT, Decayed Teeth; FS, Filled Surfaces; FT, Filled Teeth; OHI‐S, Oral Hygiene Index‐Simplified; OR, odds ratio from logistic regression models; TSF, Triceps Skinfold Thickness; WC, Waist Circumference

No significant associations were found between nutritional measures and certain variables, including prosthodontics usage, number of teeth, nor with the presence of ulcers, gingival hyperplasia or atrophic glossitis.

## 4 Discussion

This study compared oral health, hygiene, and nutritional status in IwD across institutional and family settings, revealing contrasting patterns that highlight the role of living environment in shaping distinct health risks.

Our findings indicate that institutionalized participants exhibited fewer natural teeth, poorer oral hygiene and a greater burden of untreated caries compared with their community‐dwelling peers, consistent with previous reports highlighting the vulnerability of institutionalized populations to neglected oral care [4, 5, 6, 7, 21]. In contrast, the family‐living group demonstrated significantly higher rates of treated caries, reflecting greater access to dental services, but also a markedly higher prevalence of being overweight and obese, suggesting differences in nutritional status between living environments, as suggested in previous literature [1, 2, 7, 14].

The observed differences between institutionalized individuals and those living in the community highlight the potential role of functional and behavioral status in shaping oral health outcomes. Institutionalized participants demonstrated lower levels of autonomy (Katz Index) and cooperation (Frankl scale), alongside poorer oral health, as defined above, compared to their community‐dwelling counterparts. Although functional status (Katz Index) was included as a covariate, the present study did not formally examine the independent or modifying role of functional measures in the relationship between oral health and nutritional status. Nonetheless, previous literature suggests that increased functional dependency and reduced cooperation may be associated with poorer oral health outcomes and may also have implications for nutritional status [2, 5, 6, 11, 12, 14].

Importantly, anthropometric measures in our study correlated with several oral health indices, underscoring the potential bidirectional links between oral health and systemic nutritional status, although these associations differed across living environments.

In the family‐living group, anthropometry measures demonstrated a heterogeneous pattern of associations with oral health indicators. Higher BMI was positively associated with DS, while inverse associations were observed with FT and FS. Similarly, greater waist circumference was positively associated with DS and showed a marginal positive association with DT but inversely related to restorative components (FT and FS). These findings suggest that increased adiposity may be linked with a greater burden of untreated disease while simultaneously reflecting differences in treatment history or access to restorative care. As restorative indices represent past dental interventions rather than active disease, the inverse associations with FT and FS are likely to reflect variability in care utilization or preventive practices rather than direct biological effects of adiposity [4, 5, 6, 7].

Among masticatory indicators, the number of posterior occlusal pairs showed some evidence of association with anthropometric measures. Previous studies similarly emphasize that it is functional dentition (adequate occluding pairs) and well‐fitting prostheses that sustain dietary variety and nutrient intake, not merely prosthesis usage or raw tooth counts [8, 10, 12, 20, 28, 32].

Among community‐dwelling IwD, increased TSF was associated with a higher prevalence of xerostomia and soft tissue trauma. Although previous literature suggests broader associations between nutritional status and oral mucosal health [12, 14, 15], these findings may represent novel or context‐specific associations and warrant further investigation in longitudinal studies.

Interestingly, in the institutionalized group, BMI was inversely associated with calculus and OHI‐S, while TSF was positively linked to angular cheilitis. While these findings may reflect residual confounding, previous literature suggests a plausible biological explanation. However, due to the cross‐sectional design, causal inference cannot be established, and the following interpretation should be considered exploratory and hypothesis‐generating. In our cohort, institutionalized residents reported a highly risky pattern of more frequent provision of meals, greater daily sugar intake, lower masticatory efficiency together with markedly lower toothbrushing frequency and fewer annual dental visits. Although detailed dietary composition and food texture were not formally assessed in this study, previous studies suggest that institutional dietary practices may rely on easily consumable, texture‐modified and carbohydrate‐rich foods, which may adversely affect oral health [12, 20, 22, 23]. Together, these factors may reflect a potential synergistic effect whereby repeated consumption of fermentable carbohydrates increases substrate for plaque bacteria, while poor mechanical plaque removal promotes calculus formation and caries development [13, 20]. Similar associations between dietary factors, poor oral hygiene, and oral health outcomes have been described in general, institutionalized and elderly populations [10, 21].

These findings suggest that oral hygiene practices, dietary habits, and access to preventive dental care may be important targets for intervention in institutional settings. Interventions focusing on caregiver‐supported oral hygiene, dietary management, and maintenance of functional occlusion could potentially contribute to improved oral and nutritional outcomes in this population. On the other hand, while family support may buffer against poor oral hygiene and untreated disease, it does not necessarily safeguard against dietary excess and central adiposity. This dual picture emphasizes that interventions for family dwellers should focus not only on sustaining oral hygiene practices and oral health but also on promoting balanced nutrition and prevention of overnutrition. Interdisciplinary collaboration between dentists, dietitians, caregivers and medical staff is essential to reduce the combined burdens of malnutrition and oral disease in people with disabilities no matter their living setting.

Strengths of this study include the relatively large sample size, the inclusion of both institutionalized and community‐dwelling participants and the integration of clinical dental indices with anthropometric nutritional measures. Data were collected through investigator‐administered interviews and clinical examinations performed by a hospital‐based dental professional, enhancing validity compared with caregiver‐only reporting. The consistency of associations across multiple indices supports the robustness of the findings, while the inclusion of all eligible participants across the available institutions and community settings strengthens internal and ecological validity.

Limitations include the cross‐sectional design, use of convenience sampling, restriction to one geographic region, inclusion of participants from a limited number of institutional facilities, as well as reliance on self‐reports for some behaviors. These may limit generalizability and introduce recall bias. Nutritional status was primarily evaluated through anthropometric measures, which do not fully capture dietary quality. While sugar intake and meal frequency were directly assessed in this study, other aspects of dietary composition were not directly assessed and are therefore interpreted in light of existing literature on institutional practices. Given the number of statistical tests performed, the possibility of type I error is increased, as no formal adjustment for multiple comparisons was applied.

Future research integrating detailed dietary assessment methods—such as 24‐h recalls, standardized classification of texture‐modified diets, and quantification of ultra‐processed food intake—would allow for a more comprehensive elucidation of the underlying mechanisms. Functional variables were included as covariates; however, further research is needed to explore their potential modifying role. Longitudinal studies are needed to clarify causal links between diet composition, oral hygiene and nutritional outcomes, while interventional studies could evaluate the impact of dietary modifications, caregiver training, and restoration of functional occlusion. Broader geographic sampling and integration of objective dietary measures and inflammatory biomarkers would further strengthen the evidence base.

## 5 Conclusion

This study demonstrates distinct patterns in oral and nutritional health among IwD according to their living environment. Participants residing in institutional settings presented with poorer oral hygiene and a higher burden of untreated dental caries, whereas those living in community settings were more frequently affected by overweight and obesity. The observed associations between anthropometric indicators and oral health parameters underscore the interrelated nature of nutritional status and oral health. Collectively, these findings emphasize the necessity of integrated, context‐sensitive oral and nutritional health interventions. Tailoring preventive and therapeutic strategies to the specific challenges of institutional and community living environments may contribute to improved overall health and quality of life for IwD.

## Ethics Statement

The study protocol was reviewed and approved by the Institutional Review Board of the Aristotle University of Thessaloniki (approval no.16/06.04.2022). Additional approval was obtained from the administrative boards of the three participating residential care institutions. Written informed consent was obtained from all participants or, when appropriate, from their legal guardians.

## Conflicts of Interest

The authors declare that they have no commercial or financial relationships that could be construed as a potential conflict of interest in connection with this research.

## Data Availability

The datasets generated and/or analyzed during the current study are available from the corresponding author upon reasonable request.
